# Bioengineering Tools Applied to Dentistry: Validation Methods for In Vitro and In Silico Analysis

**DOI:** 10.3390/dj10080145

**Published:** 2022-08-04

**Authors:** Jefferson David Melo de Matos, Daher Antonio Queiroz, Leonardo Jiro Nomura Nakano, Valdir Cabral Andrade, Nathália de Carvalho Ramos Ribeiro, Alexandre Luiz Souto Borges, Marco Antonio Bottino, Guilherme da Rocha Scalzer Lopes

**Affiliations:** 1 Center for Dental Biomaterials, Department of Restorative Dental Sciences, University of Florida (UF Health), Gainesville, FL 32610, USA; 2 Department of Biomaterials, Dental Materials, and Prosthodontics, Institute of Science and Technology, São Paulo State University (UNESP), São José dos Campos 12245-000, Brazil; 3 Department of Restorative Dentistry & Prosthodontics, The University of Texas Health Science Center at Houston (UTHealth) School of Dentistry, Houston, TX 77054, USA; 4 Department of Dentistry and Oral and Maxillo Facial Surgery, Universidade Federal de Juiz de Fora UFJF, Governador Valadares 36036-900, Brazil; 5 Department of Dentistry, Universidade São Francisco (USF), Bragança Paulista 12916-900, Brazil; 6 Postgraduate Program in Dentistry, Department Dentistry, University of Taubaté (UNITAU), Taubate 12080-000, Brazil

**Keywords:** finite element analysis, computing methodologies, computer simulation, dentistry

## Abstract

This study aimed to evaluate the use of bioengineering tools, finite element analysis, strain gauge analysis, photoelastic analysis, and digital image correlation, in computational studies with greater validity and reproducibility. A bibliographic search was performed in the main health databases PUBMED and Scholar Google, in which different studies, among them, laboratory studies, case reports, systematic reviews, and literature reviews, which were developed in living individuals, were included. Therefore, articles that did not deal with the use of finite element analysis, strain gauge analysis, photoelastic analysis, and digital image correlation were excluded, as well as their use in computational studies with greater validity and reproducibility. According to the methodological analysis, it is observed that the average publication of articles in the Pubmed database was 2.03 and with a standard deviation of 1.89. While in Google Scholar, the average was 0.78 and the standard deviation was 0.90. Thus, it is possible to verify that there was a significant variation in the number of articles in the two databases. Modern dentistry finds in finite element analysis, strain gauge, photoelastic and digital image correlation a way to analyze the biomechanical behavior in dental materials to obtain results that assist to obtain rehabilitations with favorable prognosis and patient satisfaction.

## 1. Introduction

Countless simulation tools in bioengineering have been used to overcome the limitations of in vitro and in silico analysis. Among them, Finite Element Analysis (FEA) and Digital Image Correlation (DIC) gain notoriety, being a procedure that analyzes dental materials, allowing a simulation with approximate characteristics of their mechanical behavior [1]. Strain Gauge Analysis (SGA) and Photoelastic Analysis (PA) is a techniques used for the experimental stress and strain analysis in mechanical structures [2]. These structures present strains under loading or the effect of temperature. Strain Gauge and Photoelastic or evaluation through strain measurement are one of the most versatile methods of evaluating mechanical behavior [3], as strain gauge sensors are widely used to measure strains in larger structures such as crowns, bones, and anatomical structures, and also associated with transducers to measure pressure, stress, force, and acceleration [2,3].

The FEA and DIC were created to solve structural engineering problems, as a study tool. Where the analysis evaluated the strain and stress degree of a solid when certain loads were applied [4]. In turn, it is based on a mathematical analysis, which consists of the fragmentation of a complex element into small elements, maintaining the same properties [5].

These elements are mathematical models described by differential equations, which are solved by computational methods to obtain the desired results [5]. The mathematical foundations of this method emerged at the end of the 18th century; however, its viability became possible only with the advance in technology, facilitating the resolution of the algebraic equations resulting from its application^6^. It is worth mentioning that its use became common during the ‘60s when it underwent a great evolution and the resource was transferred to the computer for arbitrary geometric analysis of materials that are subject to any type of load [5].

In this context, Dentistry adhered to this technique in several specialties, finding in Orthodontics great applicability of its resources [5] However, in other specialties, mainly in implant dentistry and dental prosthesis, this resource should be used with caution, since the supporting tissues are highly heterogeneous, however, when associated with other methodologies, it allows estimating the occlusal stresses generated in the surrounding bone, preventing bone resorption [6].

With this computational tool, complex biomechanical behaviors of prostheses and surrounding structures can be measured and, for analysis, it is necessary to obtain an experimental model of the object to be studied, which can be any structure of the stomatognathic system [6]. From this, a model is obtained in CAD (Computer-Aided Designer) and inserted in the finite element program ANSYSTM 19.2 (ANSYS Inc., Canonsburg, PA, USA) and, within the program, the mechanical properties and different contact conditions of the models to analyze the stress, strain, and displacements [5].

Thus, it is necessary to understand the failures that occur in the dental clinic, since in vitro studies are often not able to explain such situations, and in vivo studies have difficulties to be performed [7,8]. The use of in silico studies is an option to explain mechanical dental conditions difficult to be reproduced by other types of studies [8,9]. In this context, to gain a better understanding of biomechanical behavior in dental clinics, in silico studies are increasingly being used through bioengineering tools, such as finite element analysis [10,11]. Numerical analysis using FEA and DIC allows the simulation of loads application and provides information regarding their respective stress distribution and microstrain [12]. Whereas SGA and PA are a tool with high sensitivity that allows strain analysis through the use of strain gauge sensors [13]. In addition, the association of these two methodologies allows a correct analysis of the investigated events and helps to understand some clinical manifestations, as well as their availability to assess biomechanical behavior [14].

The present study is justified as there is in the literature an enormous variability and a discrepancy in the properties used in FEA, SGA, PA, and DIC studies. This has contributed to inaccurate searches or, at least, dubious results. Such a problem must be understood and clarified, thus eliminating a substantial bias that can compromise the final result of the study with the different tools in bioengineering applied in dentistry. Given what has been exposed, this study aims to evaluate the literature regarding the use of bioengineering tools, finite element analysis, strain gauge analysis, photoelastic analysis, and digital image correlation, in computational studies with greater validity and reproducibility.

## 2. Materials and Methods

### 2.1. Source Selection

A bibliographic search was performed in the main health databases PUBMED and Scholar Google, in which articles were published from 1955 to 2022 were collected. In the first stage, the list of retrieved articles was examined by reading the titles and abstracts. In the second stage, the studies were selected by reading the full contents. Two authors (JDMM and GRSL) performed stages 1 and 2. Experimental clinical, laboratory studies, case reports, systematic reviews, and literature reviews, which were developed on living individuals, were included. Therefore, articles that did not deal with the use of finite element analysis, strain gauge analysis, photoelastic analysis, and digital image correlation were excluded, as well as their use in computational studies with greater validity and reproducibility.

### 2.2. Data Source

Through bibliographic research, 100 articles were selected, 65 articles from PUBMED and 35 from Scholar Google (Table 1). The following titles of specific medical subjects and keywords were used: Dentistry; (DeCS/MeSH Terms), Computing Methodologies (DeCS/MeSH Terms), Computer Simulation (DeCS/MeSH Terms).

## 3. Results

According to Table 1, it can be seen that the average publication of articles in the period from 1955 to 2022 from the PubMed database was 2.03 and with a standard deviation of 1.89. While at Scholar Google, the average was 0.78 and the standard deviation 0.90. Different letters have a statistically significant difference. Thus, it is possible to verify that there was a significant variation in the number of articles, in both databases. Given the result with *p* > 0.005, there were statistically significant differences between the main health databases.

## 4. Literature Review

The difficulties found in randomized clinical studies to evaluate the biomechanical performance of materials and peri-implant tissues, since the strain and stress distribution do not allow them to be directly assessed by non-destructive means, can be overcome with the use of bioengineering tools. In this sense, finite element analysis is being used extensively in biomechanical investigations with osseointegrated implants [15]. This methodology consists of the computational mathematical analysis of a theoretical model that allows obtaining information from regions that are often inaccessible by other methods and also allows formulating consistent initial theories for the development of future research, in addition to presenting results that corroborate and support other methodologies in the same investigation [5,6,16].

The precision and applicability of the results obtained through finite element analysis are directly related to the quality of the models used in the research. Thus, the complexity of the structures involving the anatomy of the jaws and implant-supported prostheses requires the use of three-dimensional models to make the simulations similar to clinical or laboratory reality [16]. These three-dimensional models can be obtained through tools available by several CAD programs (Computer-Aided Design), scanning, or computed tomography. However, if the investigation involves a specific area, the analysis can be done in a section of the jaws to be studied and for laboratory studies. The symmetric specimens allow a faithful reproduction of the three-dimensional model, while more complex samples can also have their geometry simplified without compromising the results [5,6,17].

After obtaining the three-dimensional models, each structure must be configured according to its clinical or laboratory correspondent and for this, some information regarding the characteristics of the simulated materials is essential. To carry out the calculations of isotropic materials using the finite element method, some information is required regarding the properties of the materials, such as the elastic modulus and Poisson’s coefficient [18]. The elastic modulus or Young’s modulus (E) is a mechanical property that measures the stiffness of solid material and is defined by the stress (force per unit area) and strain (proportional strain) ratio, which is calculated by the tension (σ) and the strain (ε) ratio, therefore, E = σ/ε = (F/A)/(ΔL/Lo) [17]. The Poisson’s coefficient (v) is a dimensionless property that measures the transverse strain (about the longitudinal direction of load application), which is calculated by the ratio of the extension in the “x” direction, which is transversal, by the extension in the “z”, which is longitudinal, given by the formula, v = −εx/εz [19].

In the three-dimensional simulation of bone tissue models, the density can be calibrated according to its anatomical region, however, most studies use uniform values of elastic modulus for cortical bone with average values of 13.7 GPa, whereas medullary bone is highly variable, with mean values of 0.3 to 9.5 GPa [20,21]. For laboratory study, the simulation of bone tissue occurs through the use of polyurethane, since the material has been validated for this purpose by Miyashiro et al. (2011) [21] for presenting an elastic modulus between the cortical bone and the medullary bone, that is 3.6 GPa. The cortical bone, the medullary bone, and the polyurethane have Poisson’s coefficient with a value of 0.322. Other materials widely used in these implant restorations simulations are titanium with values of 110 GPa and 0.33 and NiCr (Nickel-Chromium) with values of 206 GPa and 0.3, for the properties of elastic modulus and coefficient Poisson’s, respectively [22,23]. In this context, three-dimensional models for the investigation of implants, components, and bone tissue or similar, allow all materials to be considered homogeneous, linear and isotropic [24].

To conclude the configuration of the three-dimensional models, the contact between the different materials must be determined. Different types of contacts are found in the literature to investigate the behavior of restorations on implants, and for linear analyzes, it can be assumed that the structures are perfectly bonded [25]. This condition is accepted based on experimental studies since, during the removal of osseointegrated implants, the fracture does not occur at the bone-implant interface [26].

On the other hand, the contacts can be considered non-linear and present a coefficient of friction between them. Previous studies used different values of friction coefficient for the contact of the medullary and cortical bone with the implant, with values of 0.65 and 0.7727, in addition to the friction coefficient for the contact between the implant and the prosthetic components, with values from 0.3 to 0.5 [17,27]. The correct configuration of the models allows for obtaining results regarding the stress distribution (tension, compression, and shear), strain, and displacement closer to the clinical or laboratory reality.

### 4.1. Measure Strain and Microstrain

There are several ways to approach finite element theory, one of the main ways to elucidate FEA, is through the Rayleigh-Ritz procedure [20]. Where it is one of the most intuitive and didactic methods, which allows the approximation of problem-solving through the principle of virtual work [28,29].

The principle of virtual work states that the total potential energy of an elastic system is minimal (or stationary) when the system is in equilibrium being the total potential energy is the sum of the gravitational potential energy and the elastic strain energy [30]. This method in turn reduces a continuous medium with infinite degrees of freedom (product of the number of nodes in a mesh by the number of unknowns per node) to a system with a finite number of degrees of freedom [31,32].

The method makes this possible, based on the hypothesis that the displacements are a function of a finite number of indeterminate coefficients that must be determined. The problem becomes the determination of these coefficients [29,33,34].

The solution to the problem is to find an expression for the potential energy of the system in terms of A the constant of the equations that describe the beam strain, differentiate this equation concerning A and equal to zero, according to the boundary conditions (Table 2) [29,30,31,32,33,34,35,36,37,38,39,40,41,42,43].

### 4.2. Finite Element Method or Analysis

A continuous element is one whose geometry is completely defined by its nodes (triangles, quadrilaterals, tetrahedrons, among others) [57]. The internal displacements of these elements are described by the displacements of the nodes using interpolation functions generally polynomial, from these functions’ expressions are obtained for the energy that must be minimized to obtain a set of algebraic equations [58]. The solutions to the equations describe the displacements in the nodes. The displacement values at each node are analogous to the values of coefficient A calculated in the example of the simply supported beam [59].

**Figure 1:**
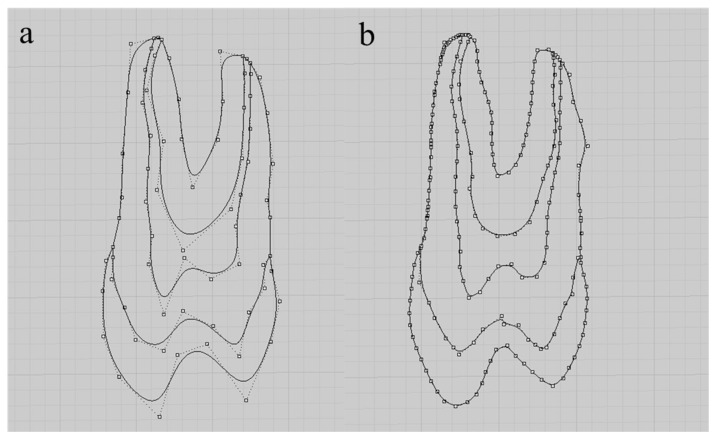
Continuous elements in two dimensions profile (2D). Legend: (**a**) row optimization; (**b**) *polyline* command.

By determining the displacements in each node, it is possible to determine the displacements and stresses in the entire continuous element. In general, displacements calculated in this way are more accurate than stresses (Figure 1).

**Figure 2:**
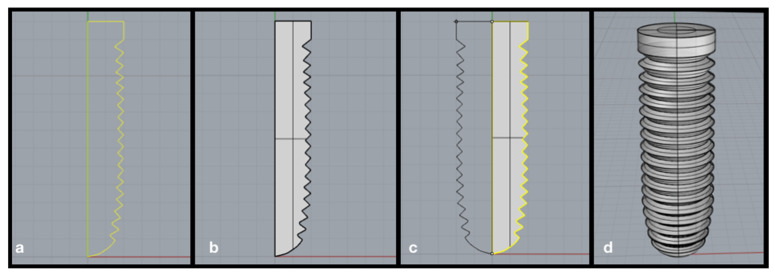
Steps to create the 3D symmetric model. Legend: (**a**) selection of lines; (**b**) surface created between the lines; (**c**) selection of the *revolve* command (*full circle*) on the “y” axis; (**d**) 3D volumetric model of the implant.

To make the results closer to the real situation, CAD software allows the creation of 3D models, and symmetric models can be performed in a simplified way. For this, we can import images using the background bitmap command, allowing lines to be traced, generating a 2D image of half of the body (e.g., dental implant). After selecting the traced lines (Figure 2a), the *planar surface* command can be used to form a surface between the lines (Figure 2b), and then the *revolve* command (*full circle*) is used with rotation about the “y” axis to form a three-dimensional model of an implant (Figure 2c,d).

To obtain models with more complex anatomies, the object can be scanned (eg prosthesis). In this case, the prosthesis was fixed on a condensation silicone base and its surface was coated with a matte spray (Cerec Optispray, Sirona, Bensheim, Germany) to facilitate the scanning process. Then the set was adjusted to the base of the extra-oral scanner (Sirona, InEos Blue, Beinsheim, Germany), allowing to obtain a “.STL” file using the software (CEREC inLab, Sirona Dental Systems, Erlanger, Germany) ensuring the faithful anatomical reproduction of a 3-element multiple prosthesis.

**Figure 3:**
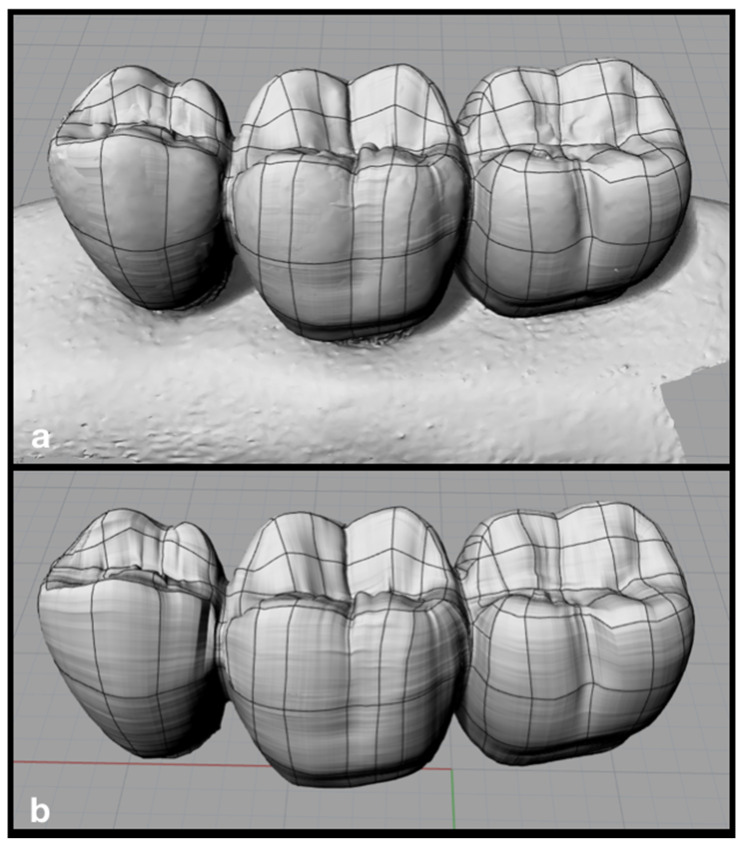
3D model of the prosthesis made from the “STL” file. Legend: (**a**) lines and meshes over the “STL” file; (**b**) 3D model of the prosthesis.

After obtaining the “.STL” file, it was exported to Rhinoceros software (version 5.4.2 SR8, McNeel North America, Seattle, WA, USA). Then, lines were drawn over the imported file using the *polylineonmesh* command (Figure 3a). The lines were then cut in all directions with the split command and simplified using the rebuild command. With the selection of four contiguous lines, several alternating surfaces were created using the networksurface command. The surface was finalized using the hide command for the lines and again the networksurface command was used between the edges of the surfaces previously created (Figure 3b).

**Figure 4:**
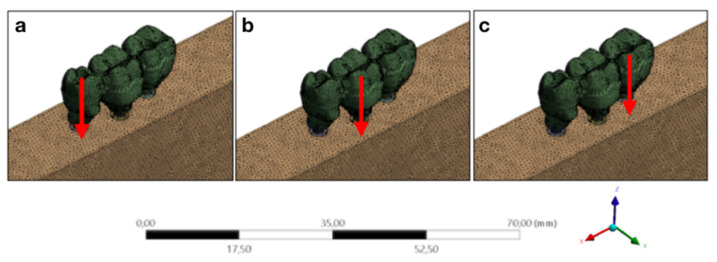
Analysis configuration for different axial loads. Legend: (**a**) Loading point A; (**b**) Loading point B; (**c**) Loading point C.

For structural static analyses, all three-dimensional models are exported to CAE software. Material properties are reported to the software, models are renamed according to what they are representing, and all structures may have different characteristics (e.g., homogeneous, isotropic, and elastic) and different contacts (e.g, bonded, coefficient of friction, smooth), trying to reproduce a condition closer to the real. Then, loads can be applied in different directions and with different intensities (Figure 4).

After the simulations, different solutions were obtained for the different structures evaluated (eg.: von Mises stress, maximum/minimum principal stress, displacement, microstrain, among others).

A large number of finite element problems can be solved using linearization as a hypothesis, where the strains are small and the behavior of the materials is considered linearly elastic [60]. The solutions are generally quick, however, there are a large number of non-linear problems in which the stresses and displacements are not proportional to the applied loads. The solutions requrigiire interactive techniques and heavy computing resources [61].

Problems involving large strains, inelastic materials, creep, plastic relaxation, hysteresis, phase transformations, and residual stresses are also addressed using nonlinear modeling [28]. Modeling involves knowledge and models that precede finite element modeling: plasticity theory, and fluency models, among others [62].

**Figure 5:**
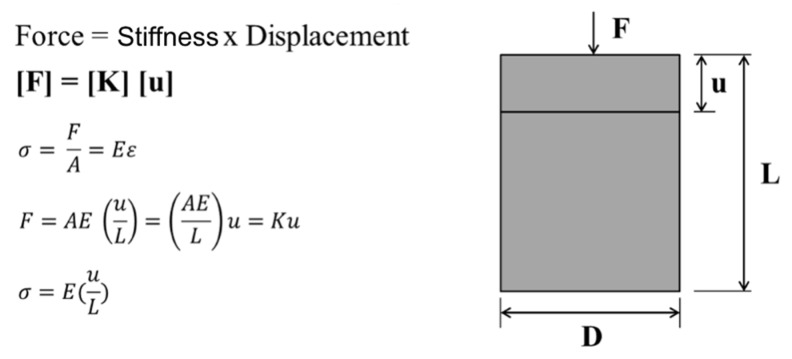
F: column of the force vectors, u: column of the displacement vectors, K: square matrix.

For static structural problems, the finite element method results in part of a complex problem transforming it into several simple equations that can be expressed in the formula (Figure 5) [29].

The finite element method employs interconnected elements, so a displacement function is attached to each finite element [29]. As far as it is concerned, each element is interconnected through common interfaces, that is, by nodes [30,31,35,36,37]. Thus, the problem can be described by an equation, where the force is equal to the stiffness times the displacement ([F] = [K] [u]) (Figure 2) [32,34,38,39].

Finite element problems can be greatly simplified by considering structures containing elements of symmetry (translation, rotation, reflection) [58]. The computational resources needed to solve a problem can be greatly reduced when using symmetry [59].

Axisymmetric symmetry is a particular case of rotational symmetry. Axisymmetric symmetry makes it possible to reduce a three-dimensional problem to a two-dimensional one [44]. When in addition to geometry there is the symmetry of load application (boundary condition and material model) it is simple to reduce the problem to the fundamental region [45].

**Figure 6:**
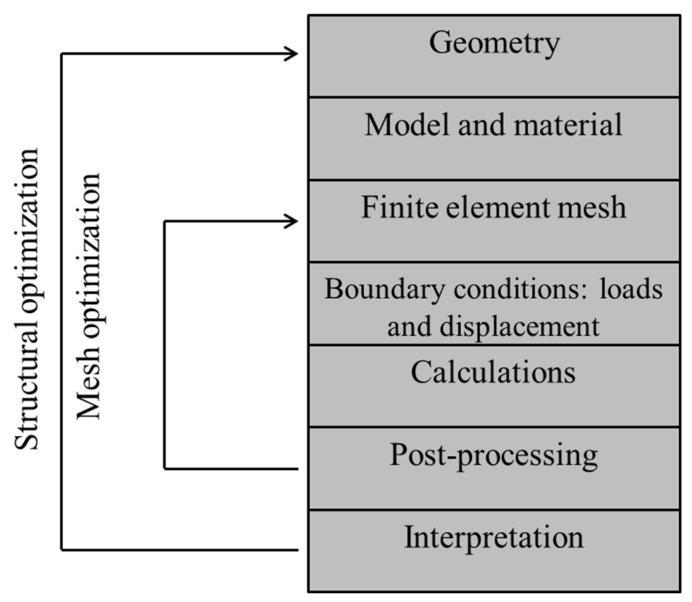
Typical finite element process.

Finite element modeling involves defining and manipulating the geometry, specifying the material and its properties, generating the finite element mesh, and defining the loads and displacements that will be applied to the component (Figure 6) [46].

In pre-processing, the properties of the materials are defined, whether they are constant or variable. Variable properties depend on time, temperature, etc., and often consume 80% of the processing time [29,44,45,46,47,48,49,50,54,63,64,65].

The results provide tables with thousands or millions of numerical values [29,50,65]. Values can be descriptive of scalar, vector, or tensor quantities. Post-processing allows for efficient interpretation of these numbers [63,64]. Color graphics, vector fields, ellipses, etc., allow easy visualization of the results obtained.

The mesh size, precision, and processing time are one of the most discussed points in the FEA, so numerous measures are developed to build a design that presents a mesh that is thin enough to give good answers, but thick enough to run without the need extraordinary computing resources [66].

When the number of nodes in the mesh is reduced, the results tend to be more inaccurate, increasing the number of nodes and the number of degrees of freedom in the mesh increases the accuracy, the number of equations, and the time required for processing [56].

### 4.3. Strain Gauge

With the increase in market competitiveness, it becomes essential that projects have reduced costs without losing the quality of their results. In this sense, the need arose to elaborate simplified methods that would allow evaluating the real conditions of material when submitted to different loads. These evaluations were based on Robert Hooke’s discovery in 1678, which became known as Hooke’s Law, which related the efforts applied to a material, through the generated stress (σ), with the resulting strain (ε), expressed by the following formula, σ = E. ε, where E is the elastic modulus [67].

Initially, essentially mechanical devices appeared that had flaws in the measurements, which proved to be a limited method. In 1843, the field of electro-electronics already showed considerable advances, a period in which Charles Wheatstone found that the effects of the variation of an electrical conductor caused by the application of mechanical stress on a material allowed the measurement of its strain. In the following decade, Wilian Thomson (1856) managed to measure such strain and, with that, several later studies allowed the development of the first electrical resistance strain gauges or Strain Gauge Analysis (SG) [68].

**Figure 7:**
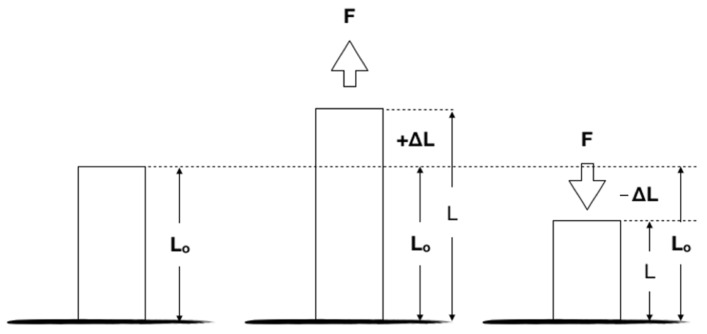
Illustration showing the positive (tensile) and negative (compression) strain of material.

The strain gauge sensors allow a high sensitivity measurement of the strain suffered by a given material (με/m) under static or dynamic loads [69,70]. This is considered an indirect measurement performed by equipment that translates variations in electrical resistance into strain levels [18,71]. Strain is described by the elongation of a section that can occur by mechanical or thermal loading and calculated by the ratio between the absolute variation in length (ΔL) and the measure of the original section (Lo), given by the formula, ε = ΔL/Lo. Therefore, compressive loads generate negative values, while tension loads generate positive values (Figure 7) [18].

**Figure 8:**
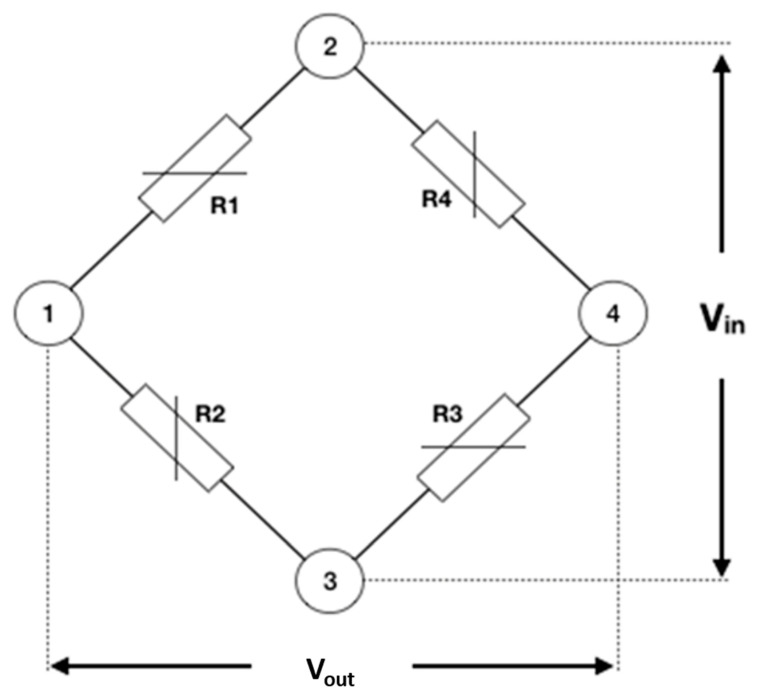
Wheatstone electrical circuit (¼ of the bridge).

The strain gauge sensors are arranged in an electrical circuit, the Wheatstone bridge, capable of measuring the variation in electrical resistance. This bridge can have different configurations, including ¼ bridge, ½ bridge, ½ diagonal bridge, and complete bridge. When ¼ of the Wheatstone bridge is used, it is formed by four resistors (R1, R2, R3, and R4) with direct or alternating voltage. If the resistance values are the same (R1 = R2 = R3 = R4) and an input voltage (Vin) and an output voltage (Vout) are connected to the circuit, there will be no potential difference (DDP) (Figure 8) [30].

In SGA, the resistance of 120 Ω or 350 Ω is normally used for stress analysis. When the strain of the material occurs, where the strain gauges are installed, there is an imbalance in the bridge, which causes some of these resistances to vary and have different values from the others. This makes the bridge unbalanced, so there is a different voltage at the output terminal, that is, a voltage variation, due to its rebalancing [18].

The variable voltage sensor has resistance (*R_g_*), while the other components are fixed value resistors [69], so the output voltage (*V_out_*) can be calculated, as shown in Formula (1).

(1) $$V_{out}=V_{in}\left(\frac{R_{3}}{R_{3}+R_{g}}-\frac{R_{2}}{R_{1}+R_{2}}\right)$$

Formula (1) is output voltage formula. Legend: *V_in_* = input voltage; *V_out_* = output voltage; *R* = resistance.

When *R_g_* is the only active strain gauge, a small variation in *R_g_* will result in an output voltage of the bridge. The gauge factor (*GF*) is defined as the ratio between the fractional change in electrical resistance and the fractional change in length (Formula (2)) [69].

(2) $$Strain\;\left(\varepsilon \right)=\frac{\left(\frac{\Delta R_{g}}{R_{g}}\right)}{GF}$$

Formula (2) is engineering strain formula. Legend: *ε* = strain; *R* = resistance; *GF* = gauge factor.

This information obtained by the strain gauges goes through a voltage amplifier and the information is obtained by a data acquisition board, normally, they are obtained as electrical voltage and expressed by the millivolt (mV) unit. These data allow to be processed and transformed in a specific quantity, for example, the microstrain (με) [71].

**Figure 9:**
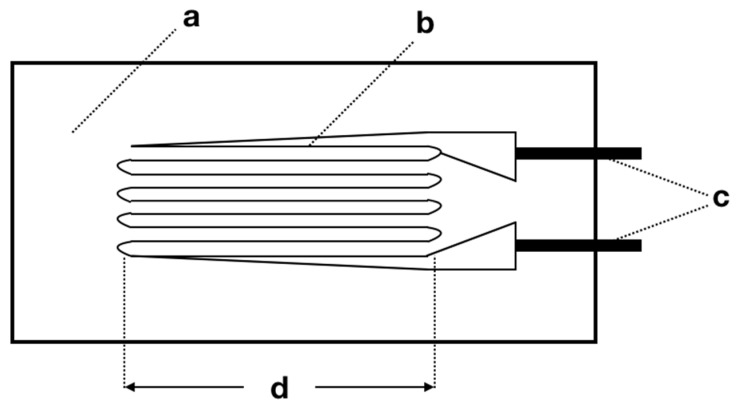
Representation of the variable resistance strain gauge unit. (**a**) support material; (**b**) measuring grid; (**c**) leads; (**d**) effective grid length.

The strain gauges are composed of support material, a measuring grid, and its leads, they can still present a variety of models and sizes for different forms of use (Figure 9).

The size of the grid does not affect the sensitivity of the strain gauges, as it measures the relative strain of material. In this sense, the use of reduced-size strain gauge sensors (approximately 1 mm) allows for the investigation of border regions, which has been used for decades as a routine methodology in research with osseointegrated implants and their structures [3,18,23,72].

**Figure 10:**
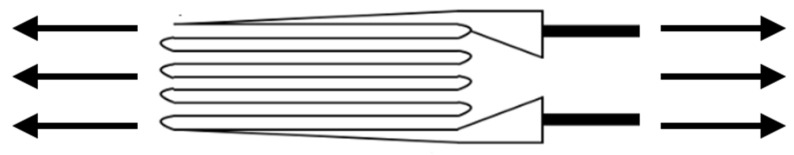
Representation of the unidirectional strain gauge sensor and the effective strain direction of the grid.

In addition, strain gauges vary their resistance according to the strain measurement grid in the effective direction, which coincides with the direction of their filaments. When the strain gauge measures only the strain in the direction of its filaments, it is considered unidirectional (Figure 10), however other models can vary its resistance when the strain is transversal to the effective direction, known as transversal sensitivity. When the direction of voltages at the measurement site is not known, strain gauges with more than one grid can be used and arranged at different angles on the same support material.

**Figure 11:**
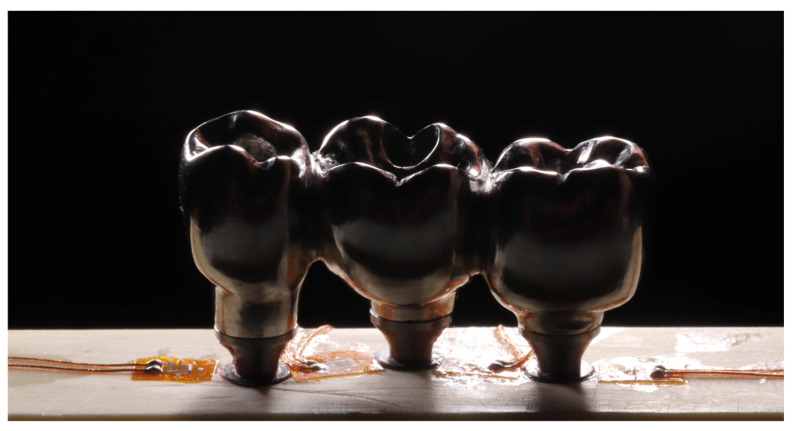
Positioning of four strain gauges on the surface of a polyurethane block.

To perform the SG tests, the terminals of each strain gauge are glued to the surface of interest and installed in an electrical signal conditioning device to record the variations in electrical resistance and transform them into a microstrain (με) (Figure 11).

The realization of previous theoretical tests, such as FEA, optimizes the laboratory investigations since it can provide information about the regions under higher stress concentration and even the direction of the stresses on the studied material. In addition, when there is a similarity that allows correlating results obtained through laboratory methodologies, such as, for example, microstrain results (με) obtained through the SGA, with results of the same magnitude obtained through theoretical models, it is possible to conclude that the theoretical models were validated, thus, other information can be obtained with considerable scientific value [14].

### 4.4. Photoelastic Analysis

The concern with achieving longevity and success with implants has brought about several types of studies, from planning bone losses and masticatory loads to the postoperative period [56]. Prosthetic planning involves several types of methods that aim to avoid these losses [73]. To better understand each patient’s condition, prosthetic plans can be simulated by direct polymerization of photoelastic materials on the patients [74]. The goal is to improve the knowledge of the stability of the implants and ensure more accuracy in this procedure, besides the prospect of giving greater longevity to the prosthesis [73,74,75].

According to Torres (2005) [73], several methods can be used to observe the stress generated between the bone and the implant, with particular notes being given to photoelastic analysis [74]. This analysis is increasingly used due to it being a simple method presenting some advantages and its effectiveness [73,74]. Photoelastic Analysis (PA) provides laboratory standards that are reliable for clinical applications. This technique uses a polariscope with a monochromatic light source, that hits the polarizer in several directions. However, only parallel wave components are transmitted [76].

Some of the advantages of PA include the joint visualization of internal stresses in bodies without the need for graphs or diagrams. It can also be applied to bodies with more complex morphology. It is limited by the requirement of models with a perfected reproduction of the original object, free of the stresses performed before the analysis [55,56,73,74,75,76].

The photoelastic analysis phenomenon was first observed in 1816 by Sir David Brewster, but it was only in 1935 that this method was introduced into dentistry by Zak. In the 1960s, PA analysis gained prominence with the aid of synthetic resins. It is initially based on the appearance of colored bands on some types of transparent materials that have been subjected to stress and also received polarized light. The bands may be evaluated over qualitative or quantitative aspects [73].

**Figure 12:**
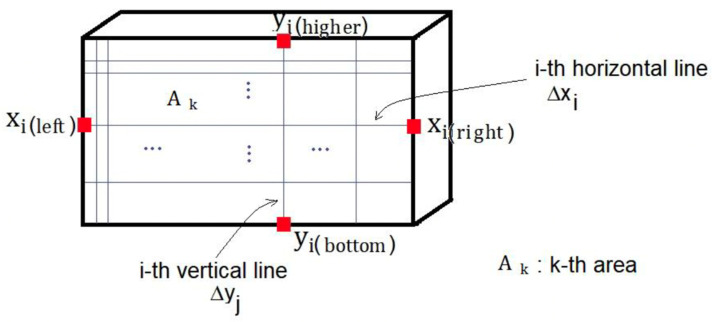
Model for capturing the distances of vertical and horizontal lines to obtain the average reference areas.

Currently, there are numerous ways of analyzing the fringe pattern adopted for each case, as well as the microstrain imposed on the surface of the material. Therefore, the main way of evaluating the strain of the material is by measuring and determining the change in the fringe pattern, this inference being allowed through the use of a fringe graph, resulting from a sequence of fringes generated with white light (Matthys, 1997). However, there are limitations in the use of this technique to evaluate the strain through comparison by a wave of fringes, given this, it was proposed by Silva et al. (2017) [34] a quantitative assessment of the fringe waves generated on the surface of the material. Since this would allow a more accurate assessment of the failure or strain pattern of the material structure. Therefore, this analysis is carried out as follows, the average reference area is defined as the product of the average horizontal length by the average vertical length of the surface of the parallelepiped from the perspective of passing light (Figure 12).

The determination of the procedure for each image is indicated from the capture of n horizontal lines and their determined lengths in pixels; then the average of the lengths of the horizontal lines and their uncertainty is calculated. After obtaining the captures, the vertical lines are traced and the lengths in pixels of the lines are determined and finally, the average of the lengths of the vertical lines and their uncertainty are calculated. Notwithstanding this, the average area is calculated from the product between the average lengths of the horizontal and vertical lines and their uncertainty.

**Figure 13:**
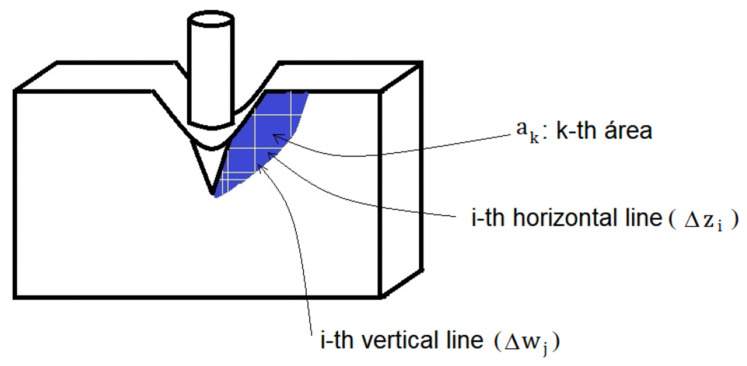
A selected area of a fringe is produced from external stress.

The average area of the fringe region is defined as the product of the average horizontal length and the average vertical length of the selected surface (Figure 13).

**Figure 14:**
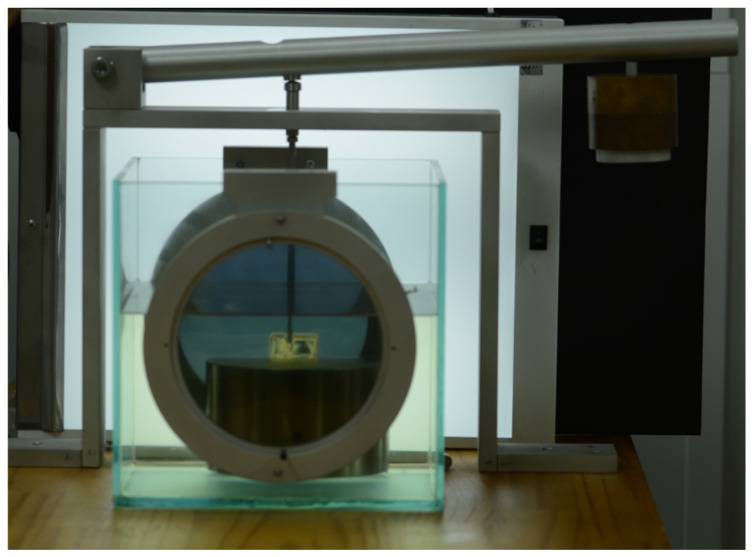
The specimen is in a polariscope device.

**Figure 15:**
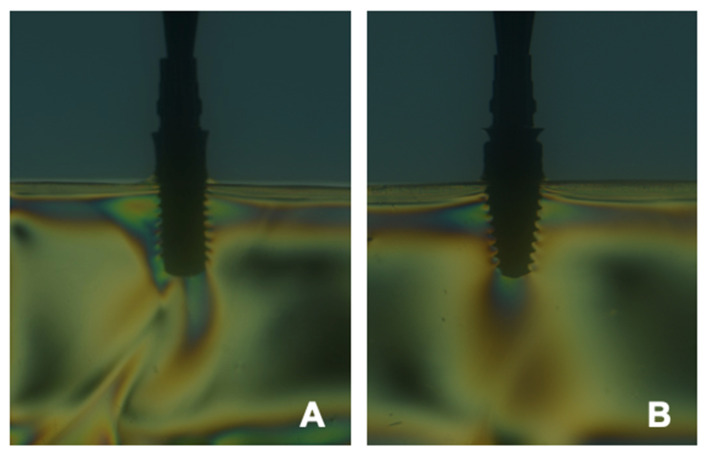
Tension fringes after loading onto abutment/implant. Legend: (**A**) Two-piece zirconia abutment/implant (Straumann PURE Two-Piece Ceramic Implant, Bone Level, Straumann Dental System Implant, Basel, Switzerland); (**B**) Two-piece titanium abutment/implant (Straumann BLT Implant, Bone Level, Straumann Dental System Implant, Basel, Switzerland).

The determination of procedures for each fringe or a fringe per image occurs by capturing n horizontal lines and determining their lengths in pixels; after that, the average of the lengths of the horizontal lines and their uncertainty is calculated. Then vertical lines are captured, and their lengths are determined in pixels; the average of the lengths of the vertical lines and their uncertainty are taken into account. Finally, the average area from the product between the average lengths of the horizontal and vertical lines and their uncertainty (Figure 14 and Figure 15).

**Figure 16:**
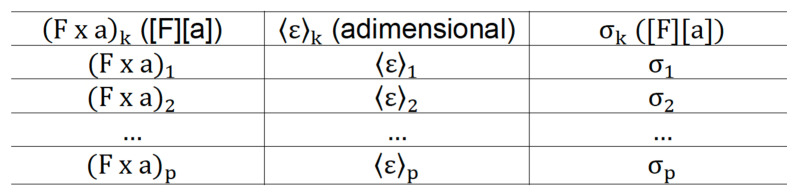
Force factor, (F × a) k, mean relative strain, 〈ε〉k, e combined uncertainty, σk.

Regarding the average relative strain from the areas, it establishes the relationship between the fringe area, 〈a〉k, by the reference area 〈A〉k-, and it is always necessary to determine the relationship between the fringe area by the reference area and its respective uncertainty, by the expressions (Figure 16).

**Figure 17:**
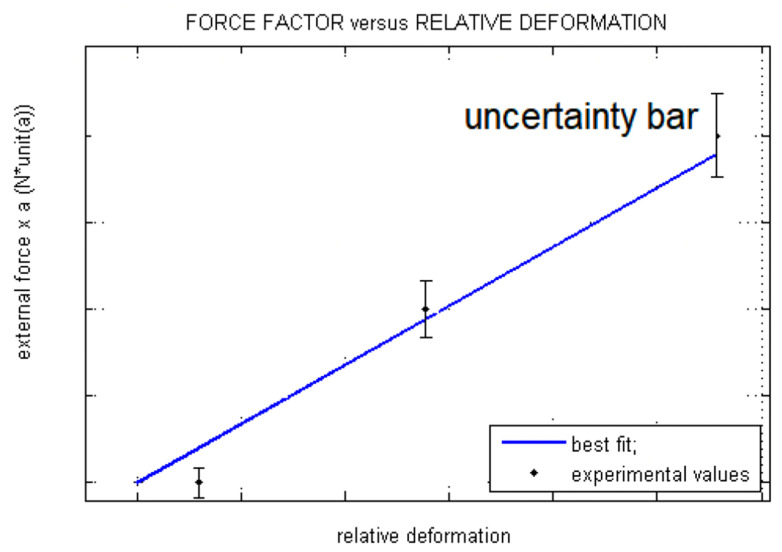
Linear regression graph of force factor by relative strain.

At the end of these initial steps, the graph of the force factor by the average strain, in which there is a transfer of uncertainty from the independent variable to the dependent variable (Figure 17).

Regarding the determination of the transfer of uncertainty from the independent variable to the dependent variable, it was done through the assumption of the adjustment function as linear. Thus, the combined uncertainties, from the uncertainties of the dependent and independent variables, using expression (8.1); then it is necessary to tabulate the values of force*factor(a), relative mean strains, and combined uncertainties; consecutively it is necessary to include the experimental values and their combined uncertainties in the graph of force*factor(a) by average strain; finally, performing the linear regression to determine the best line that represents the chosen linear function, expression (8) (Figure 15).

Torres (2005) [73] indicates that the PA model replicates the formation of bands or fringes that can be light or dark, proportionally to the differences in the main stresses that exist in the observed model. The PA technique transforms these stresses present inside the body into visible light patterns. The higher the quanta of the fringe or band, the higher the stress concentration [73,74,75].

Some studies presented in Torres (2005) [73] highlight the illustrative capacity that photoelastic analysis has in the fields of stress localization and characterization [74,75,76]. This analysis is being successfully used in research on interactions in tissue responses and physical characteristics of prosthetic restorations and implants [76]. Studies with PA verified the possibility to perceive the stress generated in the implants when there are maladjustments in these elements, together with the fact that the intensity of tightening of the implant screws did not influence the observed photoelastic patterns. and that the main purpose of PA is to evaluate the stress generated by different types of prostheses [56,73,74,75,76,77].

Torres (2005) [73] cites that the photoelastic technique was tested in an experiment where orthodontic appliances were installed in cats. There was a positive correction between the photoelastic patterns [76]. The tensile forces found in the models presented evidence of stretching of the periodontal ligament. Other research evidence resulted in the verification of periodontal fiber compression in the area of photoelastic pressure as well as the appearance of areas of hyalinization in the histological material in sites of high-stress concentration [73,74,75,76,77].

Another study developed with the aid of PA included a comparison of stresses generated in implants by pillars with different angulations [74,75]. As a complementary method, strain gauges were used. At the end of the verification, it was evident that the numerical data was similar to the visual interpretation of the photoelastic fringes which accurately illustrated the stress areas [76,77].

Given all this, it can be said that PA can be applied to check the stress generated on implants, in structures with different levels of vertical misfit, when installing two types of three-unit fixed partial dentures, to study the interactions between tissue responses, stress distribution, adaptive passivity of splinted structures, the biological behavior of implants and the relationship between the size and location of maladjustments {55,56,73–77].

### 4.5. Digital Image Correlation (DIC)

In parallel with photoelastic analysis, digital imaging in dentistry emerged in the 1980s, able to accurately analyze object strain both in 2D and 3D [51] (Figure 17). With the advent of technology, photography and computer-aided image processing techniques appeared as methods to study specific deformed objects [55,56,73]. Among the advantages of these methods, the cost stands out, being less expensive than photoelastic analysis, the Moiré method, and Holographic Interferometry [56]. One of the advantages of digital imaging is its flexibility and good adaptability [56,73].

For Beleza (2017) [51], the procedure to implement digital image correlation consists of three stages: the preparation of the specimen, the acquisition of data, and its analysis [77]. Allied to these stages we have the computer with the most suitable software [78]. This technique is not restricted to the dental area and is often used in engineering, for example [79].

According to Beleza (2017) [51], the digital image correlation (DIC) method is based on the analysis of points on a specimen’s surface using image capture. The correspondence is performed using a correlation algorithm. For this to occur accurately, a stochastic criterion is applied to the study material [78,79].

The loads applied to a certain object can cause strains that point to the properties of the material it is made of. To be sure about the size of this strain it is essential to quantify it to know the level of support and to fulfill the purpose of its creation [79]. Strains can also give information about stress distribution and stress-strain relationships to reveal underlying properties. DIC is an advantageous alternative, because, it can globally analyze the object’s strain [80].

**Figure 18:**
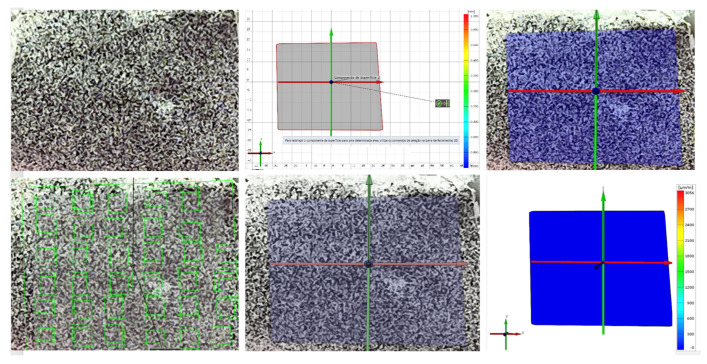
Analysis of DIC in 2D is divided into sections where they are then visualized in the next image.

For analysis, the main images are divided into sections where they are then visualized in the next image [81] (Figure 17). The acquisition of these images can be done using a digital camera or by illuminating a test sample to obtain more precise images [82] (Figure 18).

Digital images are the fundamental elements of the DIC and it needs the pixels in the correct format for the proper execution by the algorithm [78]. To be effective it needs to have good resolution, correspondence, and other parameters (internal and external) [80,81,82] (Figure 17). The internal parameters are the cost function, block size, size of the region of interest, and interpolating function. The external ones are camera resolution, illumination, stochastic pattern, acquisition frequency, and lens distortion. It is also important to properly select the software that will be used to obtain the results. They must be accurate and reliable and not require mandatory technical knowledge about their handling [79].

One of the methods adopted in digital imaging is “block correspondence”. This allows only translations of the block from one image to another [51,78,79,80]. Considering that the object can suffer tensions, cuts, tractions, and rotations, it is possible that the block can assume another form, thus not leading to incorrect correspondences [51,80].

The software used for DIC can present several features that can assist in the identification of large strains. Their analysis speed and the presentation of results can be made in color scales while pointing out possible distortions [78,79,80,81].

Therefore, the digital image correlation method has the advantage of being able to continuously evaluate stress distributions through the images resulting from the passage of time [51,78,79,80]. Both methods (PA and DIC) must obtain the model composed of different materials to serve as a reference and with the same purposes, to analyze the stresses on the implants, their biomechanical behavior, the mismatches, and strains of the object [51,78,79,80,81,82].

## 5. Discussion

In recent years, research in dentistry has been growing, this can be noted by the increase in the number of publications on the properties of dental materials and their techniques of use in different databases [83]. However, the difficulty of producing laboratory tests with relevant information can make theories unfeasible to present scientific validity [83]. In this sense, the wide use of computational methodologies is due to their high efficiency and low investment for their realization, since clinical or laboratory methodologies have their use limited by non-destructive means [84].

The three-dimensional models, used for such methodology, allow its simplification, which can generate unreliable data. Such inaccuracies are reflected in scientific production, when this information is used by other studies, generating also inaccurate results that, when used by the scientific community, produce a cascade of errors and data with questionable validity [5,6,83]. This fact has been observed in the studies of the mechanical properties of dental materials and biological structures, particularly affecting the results obtained through the FEA, SGA, PA, and DIC, which apply the elastic properties of materials obtained in the literature [11,13,76,82,83,85]. The way to assess the accuracy of these methodologies is to associate more than one experiment and verify the compatibility of their results. The compatibility values are essential to demonstrate behavior as close as possible to the real [83].

The studies that used the finite element analysis generally present tables with the values for the elastic modulus of the materials used [11,13,23,83,84,85,86,87]. The tracking of the cited references reveals the inconsistency of the information. The issue of the system of units is a problem in the literature, since the results are provided in different systems and units within each system, which makes it difficult to compare studies, and often generates erroneous transformations and error overlaps in bibliographic citations and numerical studies [83]. Especially when using SGA and PA, this type of analysis has a limitation when used for in vivo studies in which bone conditions are simulated [11,13,51,55,56,66,73,83,88,89]. It is worth mentioning that it is not possible to obtain accurate identification of the load that will be transmitted through the crown to the implant and consecutively to the bone, due to the non-fixation of the devices on the surface of the crown or implant, which may result in values lower than those established by literature [83,90,91].

When comparing the main methods of biomechanical analysis (FEA, SGA, PA, and DIC), in implants with conical connections, the authors noticed that there was a similarity in the data collected in terms of qualitative factors, however, there were differences between the methods, when evaluated on an aspect of quantification [51,55,56,65,66,73,74,75,76,77,78,79,80,81,82,83,92,93,94,95].

In this context, it can be noted that the FEA is validated by the SGA or PA, since this method assesses the stress transferred from one body to the other, thereby causing a stress distribution [83,96]. Studies show that both methods showed similar results, which may indicate the places with the highest stress concentration [83,97]. However, the FEA and PA were less sensitive than other methods of measuring stresses and are not restricted only to the polarization of translucent materials [76,83,98]. FEA is also less sensitive to environmental vibrations than the SGA, PA, and DIC [83]. In addition, the FEA can detect the movement of a body and simultaneously measure in three dimensions (mm to µm) [83,98,99,100].

However, there can be several failures in the validation of theoretical models, among them, errors in the measurements of laboratory tests, too much simplification of three-dimensional models, and areas of non-coincident loading between theoretical and laboratory models. This means that when there is a discrepancy between the results presented by the methodologies adopted the lack of compatibility and the existence of an imprecise theoretical model are evident [55]. Therefore, theoretical models can be considered validated when the failure criterion adopted is similar in approximately 10% of the results found in laboratory tests [23].

Among the limitations of theoretical simulations and laboratory tests, we can mention the absence of factors inherent to the complexity existing in the oral cavity, among them, the variation of humidity, temperature, and pH. We can also mention the use of homogeneous structures in three-dimensional models, which do not allow internal defects in their geometries. However, these limitations do not invalidate the results presented in well-designed studies but suggest caution in their interpretation and the need to associate the data presented with others available in the literature.

## 6. Conclusions

Despite the great variability of the sample used in this study, modern dentistry finds finite element analysis, strain gauge analysis, photoelastic analysis, and digital image correlation a way to analyze the biomechanical behavior in dental materials to obtain results that assist to obtain rehabilitation with favorable prognosis and patient satisfaction. In addition, the combination of two or more methods provides a more accurate description of the material’s behavior, avoiding limitations caused by the use of a single analysis method. However, further studies are needed to better understand the subject addressed in this study.

## Figures and Tables

**Table 1:** Mean ± standard deviation of the number of studies in the main health databases. CI indicates confidence interval.

Database	Mean Value ± Standard Deviation	CI 95%
Pubmed	2.03 ± 1.89 ^a^	(0.19–3.92)
Google Scholar	0.78 ± 0.90 ^b^	(0.12–1.68)

Legend: CI indicates confidence interval. Different letters indicate statistically significant differences between columns.

**Table 2:** Comparative chart for material analysis methods.

Raleigh-Ritz Method	Finite Element Method
The structure is treated as a single entity; therefore, it consists of a single element [29,30,31,35].	The structure consists of multiple elements connected by nodes [6,44,45,46,47,48,49,50].
The variables to be optimized are the coefficients A, B, C, etc., of the equations describing the problem [29,32,35,36,37,38].	Offsets and rotations are the variables to be optimized [5,6,44,46,47,51].
Less intuitive. You need to specify boundary conditions and restrictions regarding the amplitude of sine waves [29,39,40,41,42,43].	More intuitive, as the boundary conditions and restrictions refer to displacements and rotations [5,6,44,45,46,47,48,49,50,51,52,53,54,55,56].

## Data Availability

Data are available upon request.
